# Characterization of Subcutaneous Fat of Toscano Dry-Cured Ham and Identification of Processing Stage by Multivariate Analysis Approach Based on Volatile Profile

**DOI:** 10.3390/ani11010013

**Published:** 2020-12-23

**Authors:** Francesco Sirtori, Chiara Aquilani, Corrado Dimauro, Riccardo Bozzi, Oreste Franci, Luca Calamai, Antonio Pezzati, Carolina Pugliese

**Affiliations:** 1 Dipartimento di Scienze e Tecnologie Agrarie, Alimentari, Ambientali e Forestali, Università di Firenze, Scuola di Agraria, Via delle Cascine 5, 50144 Florence, Italy; chiara.aquilani@unifi.it (C.A.); riccardo.bozzi@unifi.it (R.B.); oreste.franci@unifi.it (O.F.); luca.calamai@unifi.it (L.C.); antonio.pezzati@unifi.it (A.P.); carolina.pugliese@unifi.it (C.P.); 2 Dipartimento di Agraria, Università Degli Studi di Sassari, Viale Italia 39, 07100 Sassari, Italy; dimauro@uniss.it

**Keywords:** pork, solid-phase microextraction, mass spectrometry, aroma, meat

## Abstract

**Simple Summary:**

Dry-cured ham has a characteristic flavor that originates from biochemical reactions during processing and seasoning of hams. In the case of Toscano dry-cured ham, the Protected Designation of Origin (PDO) states the minimum seasoning length in 12 months, but seasoning can be extended achieving favorable outcomes on sensory characteristics, and above all on aroma. The present study focused on subcutaneous fat of ham. Color of seasoned ham and fat composition of green and seasoned hams were studied. Special attention was paid on the study of volatile compounds, the main substances perceived by smell, present in fat. These compounds are present in large numbers, and they can be used as markers of a specific seasoning stage. For this purpose, they were analyzed by different statistical techniques to select the ones which are the most characteristic of each specific processing (0, 1, 3, 6 months) and seasoning (12, 14, 16, or 18 months) classes.

**Abstract:**

During ham processing the action of endogenous proteolytic and lipolytic enzymes leads to the development of volatile compounds (VOCs) responsible of typical aromas. Protected Designation of Origin (PDO) of Toscano ham requires at least 12 months of ripening but extended seasoning might improve flavor and economic value. This study aimed at assessing the evolution of color, fatty acids, and VOCs profile in subcutaneous fat, and, among VOCs, at identifying possible markers characterizing different seasoning length. For this purpose, a reduced pool of VOCs was selected by 3 multivariate statistical techniques (stepwise discriminant analysis, canonical discriminant analysis and discriminant analysis) to classify hams according to ripening (<12 months) or seasoning (≥12 months) periods and also to seasoning length (S12, S14, S16, or S18 months). The main VOCs chemical families steadily increased along ripening. Aldehydes and hydrocarbons reached their peaks at S16, acids and ketones remained constant from R6 to S16, whereas esters started decreasing after 12 months of seasoning. Stepwise analysis selected 5 compounds able to discriminate between ripening and seasoning periods, with 1,1-diethoxyhexane and dodecanoic acid being the most powerful descriptors for ripening and seasoning period, respectively. Instead, 12 compounds were needed to correctly classify hams within seasoning. Among them, undecanoic acid methyl ester, formic acid ethyl ester, 2,4,4-trimethylhexane, and 6-methoxy-2-hexanone had a central role in differentiating the seasoning length.

## 1. Introduction

Fat content is one of the main factors characterizing the quality of the meat. Its quantitative and qualitative characteristics affect many aspects linked both to the general quality of products and to consumer acceptability [1,2,3]. In the last years consumers have put increasing pressure on the manufacturers to produce healthier products but also with certain quality characteristics that distinguish the production process. In fact, it is important for producers to develop new lines of products or to change already existing ones in order to meet different consumer requests [3]. In the last years, Protected Designation of Origin (PDO) productions have been affected by changes in the dry-cured process due to market demands. In addition to the reduction of fat, salt, and preserving agents, there was also a request to extend the seasoning period, especially for hams. The recipe reformulation is likely to have impact on the sensory qualities and on the development of the aroma [4]. Aroma is produced by an interaction of factors including the manufacture process [5]. PDO Toscano dry-cured ham production lasts for at least 12 months. During the process numerous changes linked to water loss, salt intake, lipolysis, and proteolysis take place [6]. Sensory characteristics of the hams are mainly linked to physical and biochemical reactions caused by endogenous proteolytic and lipolytic enzymes during the drying and ripening/maturation phases [7]. The standardization of the seasoning process has meant that the qualitative variations of the products are mainly due to the intrinsic characteristics of the fresh hams [8]. With the modification of the seasoning time, chemical and sensory changes strongly depend on the duration of the ripening process [9]. An elongation of the process is generally considered a sign of high quality due to enzymatic processes which lead to an improvement of the texture and flavor of this product. Furthermore, this extension is generally linked to higher end-user prices [10]. The Canonical Discriminant Analysis (CDA) is a multivariate statistical technique which identifies a set of variables that maximizes the groups separation, whereas the Discriminant Analysis (DA) was applied to classify the samples in the different groups [11] Considering the number of variables involved, the CDA and the DA, were preferred to Principal Component Analysis (PCA) to analyze data.

The aim of this work was to study the evolution of color, fatty acids, and volatile compounds (VOCs) profile in subcutaneous fat, and, among VOCs, to identify possible markers characterizing different seasoning length.

## 2. Materials and Methods

### 2.1. Samples

In an industrial plant, thirty hams weighing 15.60 ± 1.06 kg were randomly selected and underwent the same manufacturing “Toscano” PDO Consortium manufacturing protocol, consisting of the following stages: salting (15–18 days), pre-resting (15 days), resting (60–70 days), drying (10 days), and ripening (~240 days). At the end of the ripening, hams were randomly allotted into 3 groups of 10 hams each. The first group (S14) was seasoned until 14 months, the second group (S16) until 16 months, and the third group (S18) up to 18 months. At the end of each established seasoning time, hams were dissected, and the external fat was trimmed and analyzed. Hams reached the average final weight of 10.73 ± 0.88 kg, in accordance with the PDO protocol.

Moreover, the external fat of hams belonging to group S18 was sampled along the whole processing and ripening periods to assess VOCs profile. Specifically, samples were taken at 0, 1, 3, 6, 12, 14, 16, and 18 months as described below, in paragraph 2.3 “Volatile compounds analysis”.

### 2.2. Physical and Chemical Parameters

Results on chemical composition of sliced hams, comprehensive of lean and fat, were reported in a previous research [11]. At the end of each seasoning time (14, 16, or 18 months) the trimmed fat of each sample was analyzed to assess instrumental color. As regards moisture, fat, and fatty acids profile, analysis was performed at time 0 and at the end of the seasoning time. Moisture was determined by lyophilizing to constant weight 40 g of sample, according to AOAC methods [12]. Instrumental color was assessed immediately after trimming by a Minolta Chromameter CR200 with illuminant C (Konica Minolta, Tokyo, Japan) according to CIELab coordinate system, where L* indicates lightness (or darkness), a* is the color’s position on the red-green axis and b* on the yellow-blue axis. Fatty acids were determined using a Varian GC-430 apparatus equipped with a flame ionization detector (FID) (AgilentManufacturer, Santa Clara, CA, USA) as reported by Sirtori et al. [13]. The individual methyl esters were identified by their retention time using an analytical standard (F.A.M.E. Mix, C8-C22 Supelco 18,9201AMP). Response factors based on the internal standard (C19:0) were used for quantification and results were expressed as g/100g of sample.

### 2.3. Volatile Compound Analysis

Analysis on VOCs profile were carried out by repeated sampling on each ham of group S18 (*n* = 10). Sampling took place at time 0 (R0, green ham) and after 1 (R1), 3 (R3), 6 (R6) months of ripening and at 12 (S12), 14 (S14), 16 (S16), and 18 (S18) months of seasoning. Fat was sampled using a 5-mm punch corer positioned approximately in the same location every sampling time. After each sampling, the hole was filled with a mixture of lard, salt, and pepper to prevent oxidation reactions and microbial contaminations. Subsequently, 1g of homogenized fat was grounded by liquid nitrogen and then transferred to 10 mL screw cap headspace vials adding for each sample 1 mL of distilled water and approximately 1 g of NaCl. The vials were supplemented with 40 μL of internal standard mix (ethylacetate-d8; toluene-d8; ethyl hexanoate d11; hexanoic acid d11; 3,4-dimethylphenol), either isotopologues, i.e., deuterated analogues of compounds present in the samples, added to the samples immediately before the analyses [14]. The volatile compound profile was obtained by Solid Phase Microextraction Gas Chromatography-Mass Spectrometry (SPME–GC–MS) technique. An Agilent 7890 Chromatograph (Agilent, Santa Clara, CA, USA) equipped with a 5975A MSD with EI ionisation was used for analysis. A three-phase DVB/Carboxen/PDMS 75-μm SPME fibre (Supelco, Bellafonte, PA, USA) was exposed in the head space of the vials at 60 °C for 30 min for volatile compound sampling after a 5-min equilibration time. A Gerstel MPS2 XL autosampler (GERSTEL GmbH & Co.KG, Mülheim an der Ruhr, Germany) equipped with a magnetic transportation adapter and a temperature-controlled agitator (250 rpm with on/cycles of 10 s) was used for ensuring consistent SPME extraction conditions. Chromatographic conditions were column J&W Innovax (Agilent, Santa Clara, CA, USA) 30 m, 0.25 mm, ID 0.5 μm DF; injection temperature 250 °C, splitless mode, oven program 40° for 1 min then 2 °C/min to 60 °C, then 3 °C/min to 150 °C, then 10 °C/min to 200 °C, and then 25 °C/min to 260 °C for 6.6 min. Mass spectra were acquired within the 29–350 M/Z interval with an Agilent 5975C MSD spectrometer (Agilent, Santa Clara, CA, USA) at a scan speed in order to obtain three scans/s. The identification of volatile compounds was obtained by matching the peak spectra with library spectral database and by matching of the calculated Kovats index (KI) with the KI retrieved from literature. Data are expressed as normalized area ratios with the appropriate internal standard (IS) [15].

### 2.4. Statistical Analysis

Color data were analyzed using SAS Software [16] according to the following linear model:

y_ijl_ = µ + RT_i_ + ε_ijl_ (1)

where y is the investigated variable; µ the overall mean; RT the fixed effect of processing stage; ε the random residual error. Tukey’s test with a *p*-value threshold lower than 0.05 was used to compare means.

Fat, moisture, fatty acids, and VOCs data were analyzed using SAS Software [16] according to the following linear model:

y_ijl_ = µ + RT_i_ + H_j_ + ε_ijl_ (2)

where y is the investigated variable; µ the overall mean; RT the fixed effect of processing stage (2 levels for fat, moisture, and fatty acids; 8 levels for VOCs); H the random effect of ham (with repeated measures in time); ε the random residual error. Tukey’s test with a *p*-value threshold lower than 0.05 was used to compare means.

VOCs trend during ripening and seasoning was also analyzed by SAS software (SAS Institute, Inc., Charlotte, NC, USA) applying 3 multivariate statistical techniques: stepwise discriminant analysis (SDA), CDA, and DA. The aim of multivariate analysis was to assess if there were groups of VOCs able to characterize the different ripening and seasoning times studied. Furthermore, multivariate approach was also used to outline the contribution of the identified VOCs in properly classifying hams according to their seasoning time only.

Groups separation was tested by Hotelling’s T-square test [17]. However, this test can be developed only if the pooled (co)variance matrix of data is not singular. In our research, the number of hams (rows in the matrix of data) is lower than the number of volatile compounds (columns). In this condition, any multivariate technique becomes meaningless because the (co)variance matrix does not have a full rank [18]. Therefore, a reduction of the space-variables was required. For this reason, before CDA and DA, the SDA was applied to the data to select a restricted subset of linearly independent variables, the VOCs, able to discriminate groups [19]. The obtained compounds were used in the CDA and the DA.

The CDA derives a set of new variables, called canonical functions (CAN), that are linear combination of the original compounds. For k-groups involved in the CDA, k-1 CANs are extracted. The structure of a CAN is:

(3) $$\mathrm{CAN}=c_{1}X_{1}+c_{2}X_{2}+........+c_{n}X_{n}$$

where *c_n_* are the canonical coefficients (CCs) and *Xi* are the scores of original variables. CCs indicate the partial contribution of each variable in composing the CAN. The greater the CC, the more the variable contributes to compose the CAN.

The distance between groups was evaluated by using the Mahalanobis’ distance, whereas the effective groups’ separation was tested with the corresponding Hotelling’s T-square test (De Maesschalck et al., 2000). Finally, the DA was performed to classify ham samples into seasoning groups.

The above-mentioned statistical approaches were successively applied to volatile compounds data according to the following two scenarios. In the first scenario, VOCs data were arranged in two major seasoning classes: the low maturing class (LMC) with samples belonging to 0, 1, 3, and 6 months; the high maturing class (HMC) with samples belonging to 12, 14, 16, and 18 months of seasoning. In the second scenario, only samples belonging to HMC were considered.

The discriminant procedures were applied to detect the most discriminant compounds able to correctly separate groups involved in the two scenarios. To validate the results, considering the reduced number of involved hams, the leave-one-out cross-validation technique was adopted. In practice, in each scenario, SDA, CDA, and DA were applied 10 times (being 10 the hams involved in the study) by using, at each run, one ham as validation sample. At the end, ten datasets of variables were obtained. Since compounds selected at each round could be different, the ten groups of variables were joined. The resulting compounds were used to develop the final run of CDA and DA.

## 3. Results and Discussion

### 3.1. Physical and Chemical Parameters

Subcutaneous fat is an important component of the final product, indeed, even when ham is sold in slices, fat is commonly left on. However, few studies have investigated its characteristics separately from the other section of the slice. Instrumental color parameters are shown in Table 1. According to CIELAB color values, L* and a* were affected by different seasoning times, whereas b* did not change significantly. Since b* variable is linked to yellowness and the formation of yellow-colored polymers has been associated to oxidative deterioration [20], it seems that the oxidative status of subcutaneous fat was not affected by the tested seasoning lengths. On the contrary, L* reached the greatest values as seasoning time increased, while a* showed the highest score in S16 hams and the lowest in S18 hams with S14 samples being similar to both. Analogous L* scores were reported by Tomažin et al. [21] studying the effect of sex and salting time on dry-cured ham characteristics. L* has been positively correlated to fat saturation [22], which is in accordance with the slightly higher content of saturated fatty acids (SFA) of S18 hams, as shown in Table 2. Compared to our results, higher values of a* and b* and lower of L* were observed in Iberian and Iberian × Duroc dry-cured hams reared according to two different systems [22].

Total lipids (Table 2) significantly increased moving from raw to seasoned hams, but not among different seasoning classes. The most abundant fatty acid was the oleic (C18:1), followed by palmitic (C16:0), linoleic (C18:2), and stearic (C18:0) acids, in agreement with several studies [14,23,24]. Myristic and myristoleic acids showed the highest content in S18 hams and lowest in S16 ones, with R0 and S14 hams showing intermediate amounts. Palmitic, stearic, and oleic acids showed lower content in R0 hams than in dry-cured ones, with no differences among seasoning classes. Monounsaturated fatty acids (MUFA) content was greater in seasoned than in raw hams, in accordance with oleic acid content, which is the major contributor to MUFA group in pork. This evolution was also observed by Narvàez-Rivas et al. [25] studying the changes in the fatty acid profile of subcutaneous fat of Iberian ham during dry-curing process. However, they also reported a decrease in the most important polyunsaturated fatty acids, which was not observed in the present study except for arachidonic fatty acid. Significant differences were observed only for eicosadienoic and eicosatrienoic fatty acids, but their amounts were at the lowest level in R0 samples, whereas polyunsaturated fatty acids (PUFA) showed similar amounts regardless seasoning length. Eventually, saturated fatty acids (SFA) were affected by seasoning with the lowest content in R0 hams and the highest in S12 and S18 hams, while S14 samples were similar to both. SFA are generally considered stable along processing and seasoning [25] phases, but an increase in this group was already reported in intramuscular fat of Iberian ham during the dry-curing process [26]. Probably, the decrease of moisture during processing leads to changes in sample composition and stable compounds, as SFA are detected in greater concentration.

### 3.2. Evolution of Volatile Compounds from Raw to Cured Ham

Volatile compounds develop over the ripening process, leading to the typical aroma of dry-cured products. Among the several compounds normally generated by the enzymatic and oxidative processes taking place in the tissues, aldehydes play a prominent role in characterizing dry-cured products’ aroma. Aldehydes are considered important contributors to overall aroma both for being strongly present in finished products and for their low thresholds making them easily detectable by assessors and consumers [27]. Twenty-nine aldehydes were identified in Toscano ham samples (Table 3), most of them characterized by low concentrations in green hams, whereas their presence in the cured product was considerable. The most abundant compounds in raw hams were 2-methylundecanal, 2,6-dimethyl-benzaldehyde, 2,4-dimethyl-benzaldehyde; during post-salting also pentanal and hexanal increased significantly. These two latter compounds were typical of dry-cured hams. Hexanal was reported to be the most abundant aldehyde in subcutaneous fat of Iberian ham [19,23,28], arising from linoleic acid oxidation, which is widely available in this tissue. Hexanal is also considered an important indicator of lipid oxidation; in fact, even if it contributes to the ham overall aroma with grassy and fresh notes, its excessive presence easily leads to unpleasant rancid notes and flavors [28]. Besides hexanal, also the others observed linear aldehydes originated from unsaturated fatty acids: i.e., pentanal, heptanal, octanal, and nonanal were all oxidation products of one or both oleic and linoleic acids [29]. Among linear saturated aldehydes displayed in Table 1, hexanal resulted the most abundant compound between 12 and 16 months, followed by pentanal and nonanal. These compounds reached the highest values at 16 months and drop dramatically in the last two months of ripening. This is consistent with the results reported by Andrès et al. [30], who also observed a minor peak in saturated aldehydes during the drying phase, a slight decrease at the beginning of the cellar period, probably due to further reactions with other components, and a huge increase during cellar period, that authors associated to a reduction in the activity of antioxidative systems or to a development of an intense lipolysis. Pentanal and nonanal contribute to the overall aroma with slightly fruity, nut-like notes and fatty, citrus-like notes, respectively [31]. As regard unsaturated aldehydes, 2-heptenal was the most abundant just as 2,4-heptadienal was for polyunsaturated ones. They were both characterized by green and fatty notes [31,32]. Additionally, in these groups there was an increase up to 16 months of seasoning followed by a sharp drop in the S18 hams. Probably, the cause of this decrease is to be found in the progressive disappearance of the fatty acid precursors of these aromatic compounds [33]. In fact, unsaturated aldehydes have their origin in the autoxidation of unsaturated fatty acids, in particular linoleic and linolenic acid that, in this study, although not significantly, showed a positive trend up to 14 months and then they slowly decreased.

Three branched aldehydes were observed. Two-methylbutanal and 3-methylbutanal were observed in low concentrations, but they are both considered important contributors to dry-cured ham’s aroma. Two-methyl butanal is associated with nutty, cheesy, and salty notes, while 3-methyl butanal is characterized by fruity, acorn-like, cheesy notes [31,32]. They displayed the same trend commonly described for dry-cured ham. Indeed, they showed a moderate increase in post-salting period and a deep increase during drying and cellar periods [33]. Branched aldehydes of ham were originated mainly by amino acids degradation, but there is not accordance about the pathway. Some authors postulated a microbial formation, since microorganisms are able to metabolize L-isoleucine to 2-methylbutanal and L-leucine to 3-methybutanal [33,34]; on the contrary other authors rejected this hypothesis due to the ham low microbial count, especially in the inner parts such as muscles, and postulated a non-enzymatic process via Strecker reaction [35,36], adducing the long dry-curing period as a possible alternative to high temperature in promote this kind of reaction [37]. In fat tissue, amino acids are low represented, and likely, they were quickly decomposed leading to a low concentration of branched aldehydes if compared to those usually found in muscle tissues [38,39]. Among the identified branched aldehydes, the 2-methylundecanal resulted also the most abundant volatile compound found in all samples. This compound has not been reported in dry-cured ham. It is found naturally in kumquat peel oil [31], and it is commonly used as odorant for soaps, detergents, and perfumes [40] thanks to its herbaceous, orange, fatty, and ambergris-like smell. It has also been reported in rabbit meat, but it was not classified among key odorants [41]. A recent study on interactions between protozoa and foodborne pathogenic bacteria has listed 2-methylundecanal among the VOCs originated from Listeria spp. [42], whereas European Food Safety Authority (EFSA) has defined 2-methylundecanal, among the flavoring compounds approved for addition in animal feed [43]. However, in the present study, the presence of 2-methylundecanal cannot be certainly attributed to feed rather than to microbiological metabolism or contaminant. Eventually, four aromatic aldehydes were found: benzaldehyde, benzeneacetaldehyde, 2,6-dimethylbenzaldehyde, and 2,4-dimethylbenzaldehyde. They are generally linked to amino acid degradation [44]. During the drying and the cellar periods they quickly increased until the 16th month, when together represented the 14% of the total aldehydes, then, as for the other compounds, their quantity dramatically drops. Benzaldehyde and benzeneacetaldehyde were largely reported in dry-cured ham and described as unpleasant bitter almond flower, solvent-like, fruity notes [45].

**Figure 1:**
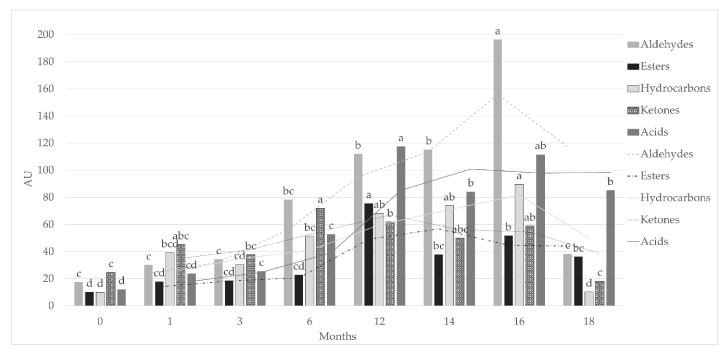
Total amounts of aldehydes, ketones, esters and hydrocarbons from R0 to S18 Toscano dry-cured hams. AU = Abundance units. Different letters (a,b,c,d) within the same chemical family indicate significant differences (*p* < 0.05) among maturing times.

Another important chemical family in dry-cured products is the esters. These compounds were generally associated to the microorganism esterase activity, but, due to the low bacterial count in ham, an alternative pathway was hypothesized. Flores et al. [46] proposed that esters could also be formed from the interaction of free fatty acids and alcohols generated by lipid oxidation in intramuscular tissues. In our study twenty esters were observed, which is a quite high number if compared with results reported by several authors [38,47]. It is worth noting that most of the studies on ham employed samples of *Biceps femoris* or *Semimembranosus* muscles, in which the fat content was very low compared to our samples. So, being the esters produced by the interaction of two lipid oxidation products, the free fatty acids and the alcohols, the greatest number of esters identified in the present work could be well explained by the matrix used. Additionally, a microbial contribution cannot be excluded being the sampling carried out on subcutaneous fat not covered by skin, where moulds and yeasts develop during the ripening and tissues are easily accessible for microbial esterase enzymes. Eventually, subcutaneous fat is also in close contact with salt used for the manufacturing, in which a considerable number of microbial communities belonging to *Micrococcaceae* was found [48]. These microorganisms were previously found in ham and associated with a significant lipolytic activity [35]. Esters developed during the ripening process, resulting thus significantly higher in finished products respect to green hams [47]. Accordingly, in the present study, the highest concentration of these compounds was observed at the end of ripening, in S12 samples (Figure 1).

Thirteen hydrocarbons were identified from R0 to S18. Hydrocarbons generally followed the trend showed by aldehydes, with an overall gradual increase until S16 and a final drop at S18. Four n-alkanes (hexane, decane, tridecane and pentadecane) were detected; they are likely products derived from lipid oxidation, as reported by several authors [49,50]. The other hydrocarbons detected are mainly branched alkanes, but two branched alkene and one branched alkyne were also identified. This chemical family is widely known in dry-cured ham, both in fat and lean matrix [39,45], but to the best of our knowledge, except for n-alkanes, none of the other compounds detected in the present study has been previously reported in literature. The identification of these compounds is likely challenging without a specific SPME fiber [51]. Despite their strong presence in dry-cured ham, most of them usually have high odor thresholds and are considered not important contributors to the aroma of dry-cured products [49]. In this study, only one aromatic hydrocarbon was found. Styrene was already reported in ham fat [39,50], its origin was alternatively reported as contaminant of plastic bags [39] or as product of degradation of phenylalanine [52], moreover styrene was associated with a penetrating odor and sweet smell [53].

Twelve ketones were found in subcutaneous fat. Three of them were aliphatic ketones (2-decanone, 2-undecanone, 2-pentadecanone). Aliphatic ketones are characteristics of dry-cured ham, being already reported by several authors both in lean and fat tissues [14,33,44,54]. 2-decanone and 2-undecanone reached their peak at S6, whereas 2-pentadecanone was related to the early ripening stages and then significantly decreased. Their aromatic notes have been described as fruity, spicy, and sometimes cheesy notes [55]. Three unsaturated ketones (1-octen-3-one, 4-hexen-2-one, 3-octen-2-one), five among polyunsaturated and methyl branched ketones (4-methyl-2-hexanone, 2,3-octanedione, 6-methyl-5-hepten-2-one, 6-methoxy 2-hexanone, 3,5-octadien-2-one), and one aromatic ketone (acetophenone) were also identified. Four-hexen-2-one is the most abundant compound for this family, it reached its maximum at 6 months of ripening then considerably declined until S18. However, at the best of our knowledge, it is the first time that this compound is reported in ham, while it was already found in pork loin and belly [56], but odor description was not reported. Concerning unsaturated and polyunsaturated ketones, 1-octen-3-one, 3-octen-2-one, 2,3-octanedione, 6-methyl-5-hepten-2-one and 3,5-octadien-2-one were already observed in fat of dry-cured ham [23,33,39]. Moreover, 1-octen-3-one was also identified as odor-active compound in ham, and described as spicy, mushroom, dirty [32]. Among the other compounds, 3-octen-2-one and 6-methyl-5-hepten-2-one have been reported as aroma active compounds in fermented meat. They were reported to have mushroom, metal and resin, pine, herbal, synthetic notes [5]. Different pathways are related to the formation of unsaturated and polyunsaturated ketones, with lipid autoxidation and microbial metabolism (β-oxidation) being the main ones [46]. Even though the microbial pathway has often been discarded for dry-cured ham due to its small internal microbial population [28], in the case of subcutaneous fat, this pathway is likely concurrent to autooxidation considering the greater exposure of sampled fat to the external environment during processing, ripening and seasoning. For instance, Andrade et al. [57], working on Iberian dry-cured ham, assumed that 2-butanone was produced by yeasts population. Accordingly, ketones resulted in being the most abundant family in early ripening phases, declining from S12 when aldehydes became the most represented group of VOCs (Figure 1).

Alcohols detected in subcutaneous fat consisted of 12 compounds. Most of them were also observed in fat [47], whole slice [30], and lean tissue [58] of Iberian ham [45] and Toscano ham [11,59]. Alcohols showed a very regular trend constantly increasing from R0 to S16 when the greatest amount for almost every compound was observed. The great increase of this family from green to seasoned fat of dry-cured ham is in accordance with results reported by Narváez-Rivas et al. [47]. After the 16th month of seasoning, they dropped at lower values. Linear and branched alcohols are known as products of lipid oxidation, whereas methyl branched ones were also linked to Strecker degradation [38]. Previous studies reported that 1-hexanol originates from palmitoleic and oleic fatty acids oxidation, while 1-octanol seems to be formed from oleic acid oxidation [60]. The most abundant alcohol observed resulted 2-octen-1-ol, followed by 1-octen-3-ol. As for many straight-chain unsaturated alcohols, they have low odor thresholds. 2-octen-1-ol is described as oily, slightly nutty and fatty waxy [31]; 1-octen-3-ol is often associated to mushroom-like, earth, fatty, and sometimes rancid notes in dry-cured products [54,61].

Carboxylic acids showed an increasing trend from green to seasoned hams. Most of the identified compounds reached the highest concentration between 12 and 16 months of seasoning and they generally decreased at S18. This is in accordance with their origin being products from hydrolysis of triglycerides and phospholipids, or from the oxidation of unsaturated fatty acids [62]. Twelve acids were identified in seasoned fat of Cinta Senese [14], however, concerning only the subcutaneous fat, few studies reported the presence of carboxylic acids. Specifically, butanoic and hexanoic acids were observed in French and Spanish dry-cured ham [19,28,39], but not in the present study. Most of the literature references about carboxylic acids in dry-cured ham refer to volatile profile of lean tissue. Nevertheless, even in this matrix, a great variability in type and number of identified compounds was observed [30,44,63,64].

Three last compounds were identified, one nitrogenous compound, one furanone, and one furan. Both furan and furanone reached their peak during the early stage of seasoning. Two-pentylfurane was detected in subcutaneous fat of Teruel white hams, Iberian hams, Spanish white hams, and French white hams [19,39]. Its trend was consistent with its origin connected to lipid oxidation [30] and in accordance with results reported for Toscano ham [11,65] and Iberian ham [58], even if these studies refer to lean matrix. Due to its quite low odor threshold, it might contribute to overall aroma by vegetable aromatic note [37]. 2(3H)dihydro-5-penthylfuranone is a lactone, it was observed only by Ruiz et al. [66] in dry-cured ham. Nevertheless, lactones have been widely reported in ham and dry-cured products with γ-butyrolactone, γ-octalactone, and γ-nonalactone being the most frequently detected in Iberian ham [45]. Similarly to 2-pentylfuran, also 2(3H)dihydro-5-penthylfuranone was likely a product of lipid oxidation of fatty acids or unsaturated aldehydes. Indeed this is considered the main origin of lactones, even if also Maillard reaction was also proposed as a possible pathway [37].

### 3.3. Prediction of the Maturing Time by a Multivariate Approach

**Figure 2:**
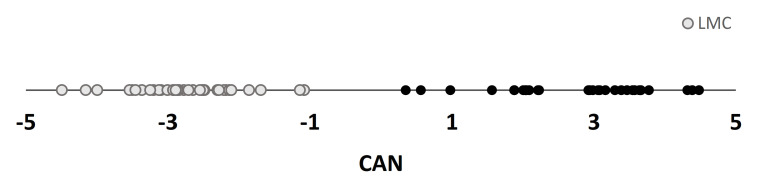
Separation of subcutaneous fat volatile compounds (VOCs) selected by SDA relatively to their capability of correctly differentiating Toscano dry-cured ham samples between low maturing classes (<12 months) and high maturing classes (≥12 months).

Several authors proposed a multivariate approach to classify dry-cured ham relying on VOCs profile. The main approach used was PCA [28,33], but also other approaches were tested, including Linear Discriminant Analysis (LDA) [33], Partial Least Square-Discriminant Analysis regression [67], and stepwise linear discriminant analysis [19,28]. In the present study three multivariate approaches were applied together to tentatively discriminate between ripening and seasoning (first scenario) and, within seasoning, to classify hams according to seasoning length (second scenario). In the first scenario, 5 compounds were selected by SDA (Table 4), Then, using the selected variables, the CDA was able to significantly (*p* < 0.001) split hams belonging to LMC (R0, R1, R3, R6) from hams belonging to HMC (S12, S14, S16, S18) (Figure 2). In details, the presence of 1,1-diethoxy-hexane was characteristics of LMC hams, whereas the other 4 compounds were related to HMC samples. Especially dodecanoic acid, with a canonical coefficient (CC) of 2.42, resulted the most characterizing compound of Toscano ham’s fat during late seasoning. Lastly, the DA correctly assigned all samples to their group of origin. Dodecanoic acid contribution in describing high maturing classes of Toscano dry-cured ham was previously observed also in *Semimembranosus* muscle (CC = 4.20) [11]. In subcutaneous fat it displayed a very clear ascending trend during ripening and reached consistent amounts in seasoning. However, it has a very high perception threshold [31], so despite being an important descriptor from the chemical point of view, it is likely not perceivable by sensorial assessment. On the contrary, to the best of our knowledge, 1,1-diethoxyhexane was not previously reported in subcutaneous fat of dry-cured products.

**Figure 3:**
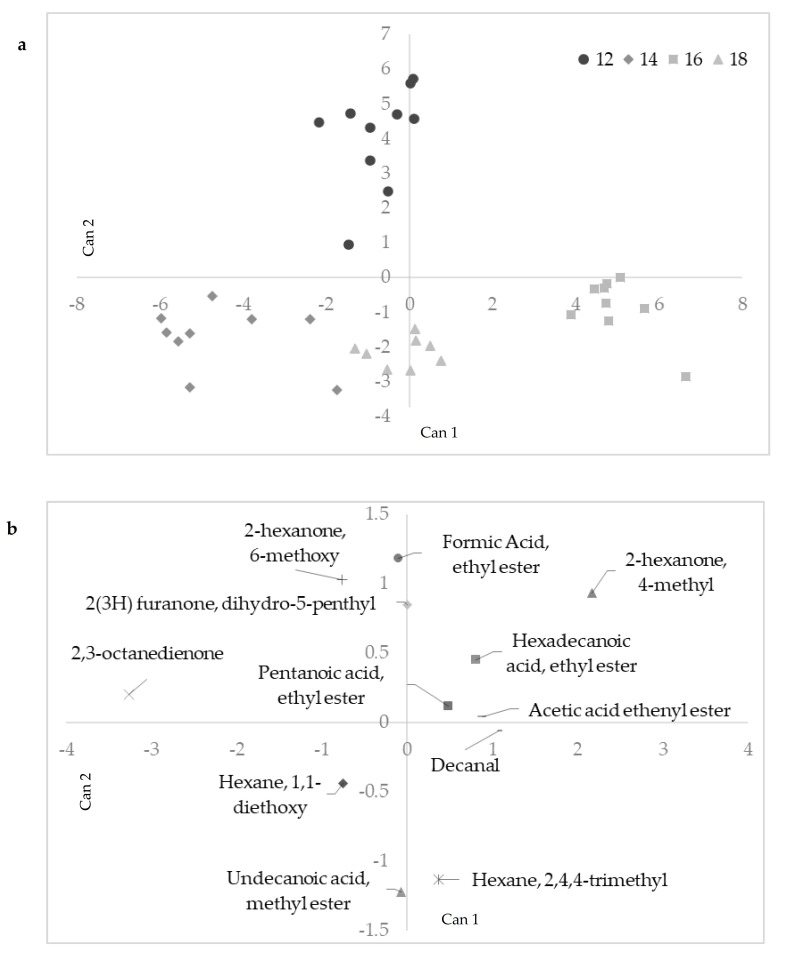
Scores (**a**) of canonical discriminant analysis within high maturing class (HMC) samples: S12, S14, S16, and S18 months of seasoning. (**b**) Loadings of canonical discriminant analysis within HMC.

In the second scenario, 12 VOCs were identified by SDA to discriminate samples into seasoning classes (Table 5) and then used to correctly assign samples to each group. The first and second canonical functions accounted for the 87% of the total variance and they were able to highlight differences among groups (Figure 3a). Can1 separated S12, S14, and S18 groups from S16. Among the compounds that weighed the most in Can1 there were 3 ketones (2,3-octanedione, 4-methyl-2-hexanone, and 6-methoxy-2-hexanone) and 1 aldehyde (decanal) (Figure 3b). Can2 separated samples belonging to S12 from the other groups. In this case, the most important compounds were 2 esters (formic acid ethyl ester and undecanoic acid, methyl ester), 1 hydrocarbon (2,4,4-trimethylhexane), and 1 ketone (6-methoxy-2-hexanone). 2,3-octanedione, 4-methyl-2-hexanone and decanal resulted in the highest CCs of Can1. Among them, special importance in overall aroma of dry-cured ham is attributed to 2,3-octanedione, which has a “warmed-over” flavor [31]. Moreover, decanal, which originates from autoxidation of oleic fatty acid, was already identified among the main descriptors to characterize samples from different dry-curing periods by LDA [33]. According to Figure 3a,b, this compound, together with 4-methyl-2-hexanone, were mainly involved in the discrimination of S16 from S12, S14, and S18. Focusing on Can2, 4 compounds had CCs higher than 1. These compounds were: undecanoic acid methyl ester (−1.22), formic acid ethyl ester (+1.18), 2,4,4-trimethylhexane (−1.13) and 6-methoxy-2-hexanone (+1.03). According to Figure 3a, formic acid ethyl ester and 6-methoxy-2-hexanone were linked to S12 hams, whereas undecanoic acid methyl ester and 2,4,4-trimethylhexane resulted to be good descriptors of hams seasoned for more than 12 months. In comparison with the previous study on VOCs of *Semimembranosus* muscle [11], a lower number of VOCs were needed to correctly classify samples according to their actual ripening and seasoning stage. This suggests that, in subcutaneous fat, there are compounds that could be powerful markers for assessing processing stages of hams.

## 4. Conclusions

In conclusion, instrumental color of subcutaneous fat was affected by time, especially L* score was higher for longer seasoning time, partially in agreement with the greater degree of saturation observed in the higher maturing classes. Oleic acid, the main contributor to MUFA amount, showed no difference among seasoning groups. According to VOCs profile, almost every identified compound was affected by ripening and seasoning times. Regardless of single compounds, the main chemical families steadily increased until R6, then different trends were observed. Aldehydes and hydrocarbons reached their peaks at S16, ketones and acids instead showed the highest total content at R6 and R12, respectively. Lastly, esters started to decrease after 12 months of seasoning. Moreover, at S18 most of the main compounds involved in dry-cured ham overall aroma already declined to value similar to ripening phases. In the future, it would be interesting to thoroughly investigated also the enzymatic activity taking place during the different processing stages. The multivariate approach adopted highlighted the importance of 5 compounds present in subcutaneous fat to discriminate between ripening and seasoning stages (1,1-diethoxyhexane, 3-methyl-ethyl ester butanoic acid, 2,4-dimethyl-benzaldehyde, butanoic acid ethyl ester, dodecanoic acid). Instead, 12 compounds were selected to classify hams according to seasoning length. Among them, 4 VOCs with CCs > 1 (undecanoic acid methyl ester, formic acid ethyl ester, 2,4,4-trimethylhexane, and 6-methoxy-2-hexanone) had a central role in differentiating the clusters.

## Figures and Tables

**Table 1:** Instrumental color of subcutaneous fat trimmed from Toscano dry-cured ham.

	Seasoning			RMSE ^1^	*p*
	S14 (*n* = 10)	S16 (*n* = 10)	S18 (*n* = 10)		
Instrumental color					
L*	76.125 ^b^	77.948 ^a,b^	79.068 ^a^	1.923	0.0178
a*	2.804 ^a,b^	3.763 ^a^	2.238 ^b^	1.086	0.0302
B*	3.964	3.192	4.158	0.912	0.0869

^1^ Root mean square error. Different letters (a,b) within the same chemical family indicate significant differences (*p* < 0.05) among maturing times.

**Table 2:** Moisture and fatty acids profile of raw and seasoned (S14, S16, S18) subcutaneous fat of Toscano dry-cured ham.

Seasoning					RMSE ^1^	*p*
	R0 (*n* = 30)	S14 (*n* = 10)	S16 (*n* = 10)	S18 (*n* = 10)		
Moisture (%)	16.955 ^a^	2.941 ^b^	3.161 ^b^	2.744 ^b^	7.288	<0.0001
Total lipids	68.423 ^b^	76.718 ^a^	76.246 ^a^	78.091 ^a^	4.774	<0.0001
C12:0	0.051	0.0386	0.025	0.027	0.029	0.0343
C14:0	0.420 ^a,b^	0.434 ^a,b^	0.409 ^b^	0.459 ^a^	0.040	0.0366
C14:1-n5	0.005 ^a,b^	0.005 ^a,b^	0.004 ^b^	0.005 ^a^	0.001	0.0055
C15:0	0.011	0.011	0.010	0.013	0.002	0.1094
C16:0	5.599 ^b^	6.103 ^a^	5.942 ^a,b^	6.460 ^a^	0.462	<0.0001
C16:1	0.893	0.928	0.902	0.990	0.102	0.0992
C17:0	0.064	0.066	0.068	0.078	0.014	0.1070
C17:1	0.067	0.067	0.067	0.079	0.015	0.1955
C18:0	2.326 ^b^	2.628 ^a^	2.624 ^a^	2.745 ^a^	0.235	<0.0001
C18:1	11.289 ^b^	12.578 ^a^	12.253 ^a^	12.959 ^a^	0.041	<0.0001
C18:2-n6cis	2.962	3.210	3.167	2.966	0.404	0.2549
C18:3-n3	0.203	0.217	0.215	0.206	0.022	0.2212
C20:0	0.047	0.051	0.0471	0.052	0.007	0.1340
C20:1	0.270	0.296	0.276	0.295	0.036	0.2295
C20:2-n6	0.157 ^b^	0.187 ^a^	0.181 ^a,b^	0.164 ^a,b^	0.028	0.0126
C20:3-n6	0.027	0.025	0.023	0.022	0.006	0.0697
C20:4-n6	0.062 ^a^	0.059 ^a,b^	0.058 ^a,b^	0.050 ^b^	0.010273	0.0235
C20:3-n3	0.090 ^b^	0.104 ^a^	0.102 ^a,b^	0.103 ^a,b^	0.014215	0.0111
C22:4-n6	0.087	0.105	0.106	0.103	0.022764	0.0714
C22:5-n3	0.081	0.097	0.097	0.096	0.020967	0.0624
C20:5-n3	0.000	0.009	0.000	0.009	0.015231	0.2337
SFA	8.521 ^b^	9.333 ^a^	9.126 ^a,b^	9.834 ^a^	0.694	<0.0001
MUFA	12.524 ^b^	13.873 ^a^	13.503 ^a^	14.329 ^a^	0.994	<0.0001
PUFA	3.671	4.013	3.945	3.720	0.479	0.1671
PUFA-n6	3.297	3.587	3.531	3.305	0.439	0.2058
PUFA-n3	0.320	0.355	0.340	0.340	0.039	0.0725

^1^ Root mean square error. Different letters (a,b) within the same chemical family indicate significant differences (*p* < 0.05) among maturing times.

**Table 3:** Effect of ripening and seasoning times on volatile compounds of subcutaneous fat Toscano dry-cured ham (*n = 10*).

Volatile Compound	KI ^a^	ID ^b^	RMSE ^c^	Time								p
				R0	R1	R3	R6	S12	S14	S16	S18	
Aldehydes												
2-methylbutanal	880	MS/KI	0.05	0.02 ^c^	0.01 ^c^	0.03 ^c^	0.11 ^b^	0.16 ^ab^	0.13 ^ab^	0.19 ^a^	0.11 ^b^	<0.0001
3-methylbutanal	884	MS/KI	0.05	0.01 ^e^	0.02 ^e^	0.05 ^d e^	0.12 ^bcd^	0.19 ^ab^	0.14 ^abc^	0.21 ^a^	0.09 ^cde^	<0.0001
Pentanal	974	MS/KI	2.28	0.15 ^c^	1.40 ^bc^	2.12 ^bc^	1.42 ^bc^	8.27 ^a^	2.50 ^bc^	3.78 ^b^	1.07 ^bc^	<0.0001
Hexanal	1081	MS/KI	3.23	0.17 ^c^	1.90 ^c^	3.05 ^bc^	4.03 ^bc^	8.59 ^a^	7.42 ^ab^	11.72 ^a^	2.24 ^c^	<0.0001
Heptanal	1183	MS/KI	0.26	0.01 ^d^	0.18 ^d^	0.30 ^d^	0.28 ^d^	1.04 ^ab^	0.70 ^bc^	1.22 ^a^	0.31 ^cd^	<0.0001
Octanal	1287	MS/KI	0.73	0.04 ^b^	0.08 ^b^	0.13 ^b^	0.22 ^b^	0.53 ^b^	2.22 ^a^	2.00 ^a^	0.08 ^b^	<0.0001
2-Heptenal	1318	MS/KI	2.92	0.07 ^c^	0.33 ^c^	1.37 ^c^	3.94 ^bc^	7.32 ^ab^	8.06 ^ab^	10.80 ^a^	3.70 ^bc^	<0.0001
Nonanal	1392	MS/KI	0.54	0.05 ^c^	0.18 ^c^	0.34 ^c^	0.72 ^bc^	1.29 ^b^	1.38 ^ab^	2.11 ^a^	0.41 ^c^	<0.0001
2,4 hexadienal	1402	MS/KI	0.69	0.04c	0.01 ^c^	0.18 ^c^	0.69 ^bc^	1.26 ^ab^	1.61 ^ab^	2.11 ^a^	0.59 ^bc^	<0.0001
2-octenal	1442	MS/KI	0.70	0.02 ^c^	0.05 ^c^	0.27 ^c^	1.53 ^ab^	1.39 ^b^	1.46 ^ab^	2.45 ^a^	0.59 ^bc^	<0.0001
2,4 heptadienal	1493	MS/KI	2.38	0.16 ^d^	0.24 ^d^	0.28 ^cd^	1.29 ^cd^	3.41 ^bc^	5.82 ^ab^	8.22 ^a^	1.82 ^cd^	<0.0001
Decanal	1498	MS/KI	0.13	0.01 ^b^	0.02 ^b^	0.02 ^b^	0.09 ^ab^	0.04 ^b^	0.03 ^b^	0.23 ^a^	0.03 ^b^	0.008
2,4-Heptadienal (E,E)-	1501	MS/KI	2.80	0.15 ^c^	0.11 ^c^	0.58 ^c^	2.19 ^bc^	5.54 ^b^	6.25 ^b^	10.70 ^a^	2.43 ^bc^	<0.0001
Benzaldehyde	1515	MS/KI	0.68	0.01 ^d^	0.14 ^cd^	0.28 ^cd^	0.85 ^bcd^	1.03 ^bc^	1.56 ^d^	2.82 ^a^	0.66 ^bcd^	<0.0001
2-nonenal	1532	MS/KI	0.97	0.00 ^c^	0.06 ^c^	0.30 ^bc^	1.36 ^bc^	1.38 ^bc^	1.62 ^b^	3.43 ^a^	0.70 ^bc^	<0.0001
2-methylundecanal	1644	MS/KI	18.86	11.68 ^b^	15.30 ^b^	13.64 ^b^	23.42 ^b^	29.19 ^ab^	32.14 ^ab^	51.96 ^a^	9.25 ^b^	<0.0001
2-Dodecenal	1844	MS/KI	0.93	0.03 ^d^	0.00 ^cd^	0.22 ^cd^	0.90 ^bcd^	1.72 ^b^	1.39 ^bc^	3.51 ^a^	0.62 ^bcd^	<0.0001
Benzeneacetaldehyde	1646	MS/KI	1.72	0.01 ^d^	0.07 ^d^	0.20 ^d^	1.95 ^bcd^	1.44 ^cd^	4.02 ^ab^	6.07 ^a^	3.85 ^abc^	<0.0001
trans, trans-nona-2,4-dienal	1704	MS/KI	2.52	0.12 ^c^	0.04 ^c^	0.51 ^c^	2.74 ^bc^	4.05 ^b^	4.80 ^b^	9.13 ^a^	1.36 ^bc^	<0.0001
2-undecenal	1717	MS/KI	1.00	0.04 ^c^	0.04 ^c^	0.18 ^c^	0.74 ^bc^	1.72 ^b^	1.40 ^a^	3.73 ^bc^	0.51 ^bc^	<0.0001
2,4 decadienal	1797	MS/KI	3.49	0.17 ^b^	0.23 ^b^	0.24 ^b^	1.48 ^b^	2.21 ^b^	4.67 ^b^	10.50 ^a^	0.93 ^b^	<0.0001
2,6-dimethylbenzaldehyde	1640	MS/KI	4.20	2.98 ^d^	5.55 ^bcd^	4.46 ^cd^	10.29 ^abc^	11.81 ^ab^	8.13 ^abcd^	13.59 ^a^	2.39 ^d^	<0.0001
2,4-dimethylbenzaldehyde	1742	MS/KI	4.21	2.98 ^c^	5.55 ^bc^	4.47 ^c^	10.43 ^ab^	11.81 ^ab^	8.13 ^abc^	13.60 ^a^	2.39 ^c^	<0.0001
Tetradecanal	1910	MS/KI	0.17	0.12	0.13	0.17	0.27	0.11	0.08	0.05	0.14	0.207
Pentadecanal	2042	MS/KI	0.15	0.25 ^ab^	0.38 ^a^	0.23 ^ab^	0.13 ^d^	0.23 ^ab^	0.04 ^b^	0.07 ^b^	0.03 ^b^	<0.0001
Octadecanal	2400	MS/KI	0.04	0.02 ^e^	0.04 ^de^	0.04 ^de^	0.10 ^cd^	0.21 ^a^	0.13 ^bc^	0.18 ^ab^	0.15 ^bc^	<0.0001
Butanal	867	MS/KI	0.13	0.04 ^c^	0.18 ^bc^	0.21 ^bc^	0.27 ^ab^	0.40 ^a^	0.14 ^bc^	0.18 ^bc^	0.07 ^c^	<0.0001
Esters												
Formic Acid, ethyl ester	825	MS/KI	2.90	1.99 ^c^	6.57 ^b^	5.02 ^bc^	3.11 ^bc^	11.75 ^a^	3.54 ^bc^	4.54 ^bc^	2.84 ^bc^	<0.0001
Acetic acid ethenyl ester		MS	0.30	0.12 ^d^	0.13 ^d^	0.26 ^cd^	0.32 ^bcd^	1.07 ^a^	0.34 ^bcd^	0.69 ^ab^	0.62 ^bc^	<0.0001
Butanoic acid, ethyl ester	1037	MS/KI	0.39	0.09 ^b^	0.48 ^b^	0.50 ^b^	0.14 ^b^	1.17 ^a^	0.52 ^b^	0.62 ^b^	1.48 ^a^	<0.0001
Butanoic acid, 3-methyl-, ethyl ester	1066	MS/KI	0.20	0.02 ^b^	0.03 ^b^	0.06 ^b^	0.03 ^b^	0.14 ^ab^	0.08 ^b^	0.10 ^ab^	0.40 ^a^	0.009
Pentanoic acid, ethyl ester	1142	MS/KI	0.12	0.01 ^d^	0.04 ^d^	0.06 ^d^	0.07 ^cd^	0.49 ^a^	0.33 ^ab^	0.41 ^ab^	0.24 ^bc^	<0.0001
Heptanoic acid, ethyl ester	1332	MS/KI	0.53	0.19 ^c^	0.42 ^c^	0.37 ^c^	0.62 ^bc^	2.18 ^a^	0.94 ^bc^	1.26 ^b^	0.28 ^c^	<0.0001
Octanoic acid, ethyl ester	1441	MS/KI	2.85	1.28 ^d^	1.97 ^d^	2.63 ^d^	2.16 ^d^	11.78 ^a^	4.27 ^cd^	7.43 ^bc^	10.09 ^ab^	<0.0001
Nonanoic acid, ethyl ester	1541	MS/KI	0.13	0.29 ^ab^	0.19 ^abc^	0.15 ^abc^	0.05 ^c^	0.33 ^a^	0.13 ^bc^	0.25 ^ab^	0.10 ^bc^	0.0002
Decanoic acid, methyl ester	1581	MS/KI	0.16	0.03 ^c^	0.03 ^c^	0.03 ^c^	0.13 ^bc^	0.43 ^a^	0.33 ^ab^	0.42 ^a^	0.42 ^a^	<0.0001
Decanoic acid, ethyl ester	1643	MS/KI	2.09	0.71 ^b^	0.44 ^b^	0.68 ^b^	0.70 ^b^	6.31 ^a^	3.32 ^ab^	1.11 ^b^	2.49 ^b^	<0.0001
Benzoic acid, ethyl ester	1645	MS/KI	0.04	0.04 ^cd^	0.05 ^cd^	0.03 ^d^	0.08 ^bcd^	0.13 ^ab^	0.10 ^abc^	0.15 ^a^	0.06 ^cd^	<0.0001
Phthalic acid, butyl 1-cyclopentylethyl ester		MS	2.48	2.46 ^a^	2.27 ^a^	2.06 ^a^	1.16 ^b^	1.15 ^b^	0.21 ^c^	0.38 ^c^	0.51	<0.05
Hexadecanoic acid, ethyl ester	2243	MS/KI	0.81	0.27 ^c^	0.11 ^c^	0.31 ^c^	0.52 ^c^	3.29 ^a^	1.16 ^bc^	2.73 ^a^	2.20 ^ab^	<0.0001
Undecanoic acid, methyl ester		MS	4.14	1.00 ^b^	1.16 ^b^	1.79 ^b^	5.10 ^ab^	5.67 ^ab^	5.78 ^ab^	7.72 ^a^	3.36 ^ab^	0.005
Heptanoic acid, ethyl ester	1331	MS/KI	0.53	0.19 ^c^	0.42 ^c^	0.37 ^c^	0.62 ^bc^	2.18 ^a^	0.93 ^bc^	1.27 ^b^	0.28 ^c^	<0.0001
Octanoic acid, methyl ester	1437	MS/KI	0.21	0.02 ^c^	0.03 ^c^	0.06 ^c^	0.18 ^bc^	0.44 ^ab^	0.38 ^ab^	0.42 ^ab^	0.63 ^a^	<0.0001
Succinic acid, 3,3-dimethylbut-2-yl isobutyl ester		MS	3.50	0.53 ^b^	0.70 ^b^	1.30 ^b^	2.81 ^b^	3.03 ^ab^	5.14 ^ab^	7.84 ^a^	1.18 ^b^	0.0002
4-Decenoic acid, ethyl ester, (Z)-	1699	MS/KI	0.21	0.06 ^b^	0.07 ^b^	0.11 ^b^	0.19 ^b^	0.79 ^a^	0.34 ^b^	0.79 ^a^	0.80 ^a^	<0.0001
Dodecanoic acid, ethyl ester		MS	0.54	0.05 ^c^	0.03 ^c^	0.05 ^c^	0.23 ^bc^	0.62 ^abc^	0.50 ^abc^	1.16 ^a^	1.03 ^ab^	<0.0001
Hydrocarbons												
Hexane	600	MS/KI	0.26	0.11 ^d^	0.20 ^cd^	0.27 ^bcd^	0.20 ^cd^	0.83 ^a^	0.49 ^abcd^	0.60 ^ab^	0.51 ^abc^	<0.0001
2,2,3,3-tetramethylbutane		MS	0.84	0.23 ^b^	0.47 ^b^	0.46 ^b^	1.00 ^ab^	1.74 ^a^	1.07 ^ab^	1.09 ^ab^	0.95 ^ab^	0.086
1-butoxy-2-ethyl-1-hexene		MS	14.77	8.08 ^bc^	30.00 ^ab^	20.61 ^abc^	8.54 ^bc^	36.31 ^a^	7.52 ^bc^	9.72 ^bc^	4.80 ^c^	<0.0001
Decane	1000	MS/KI	12.41	0.26 ^b^	3.10 ^b^	1.11 ^b^	27.69 ^a^	14.05 ^ab^	7.37 ^b^	7.29 ^b^	-0.41 ^b^	<0.0001
2,3-dimethylpentane	668	MS/KI	3.47	0.21 ^c^	2.18 ^c^	3.39 ^bc^	4.41 ^bc^	9.45 ^a^	8.29 ^ab^	13.16 ^a^	2.48 ^c^	<0.0001
1,1-diethoxyhexane	1235	MS/KI	1.44	1.90 ^bc^	2.87 ^abc^	3.23 ^ab^	4.76 ^a^	2.46 ^bc^	1.22 ^bc^	1.65 ^bc^	0.83 ^c^	<0.0001
Tridecane	1300	MS/KI	0.22	0.00 ^b^	0.04 ^ab^	0.01 ^b^	0.37 ^a^	0.03 ^b^	0.02 ^b^	0.05 ^ab^	0.00 ^b^	0.007
Styrene	1254	MS/KI	0.05	0.01 ^e^	0.05 ^de^	0.03 ^de^	0.21 ^a^	0.15 ^ab^	0.10 ^bcd^	0.12 ^bc^	0.05 ^cde^	<0.0001
2,4,4-trimethylhexane,		MS	13.19	0.54 ^b^	−0.79 ^b^	0.07 ^b^	0.24 ^b^	0.39 ^b^	44.87 ^a^	53.18 ^a^	−0.67 ^b^	<0.0001
Pentadecane	1500	MS/KI	0.08	0.01 ^b^	0.13 ^ab^	0.10 ^ab^	0.14 ^a^	0.04 ^ab^	0.02 ^b^	0.04 ^ab^	0.01 ^b^	<0.0001
4-methyl-3-nonen-5-yne		MS	1.42	0.11	0.20	0.23	1.64	0.26	0.26	0.07	0.31	0.274
1-Oxa-3,4-diazacyclopentadiene		MS	0.37	0.01 ^c^	0.12 ^bc^	0.21 ^bc^	0.15 ^bc^	0.88 ^a^	0.56 ^ab^	0.76 ^a^	0.59 ^ab^	<0.0001
3-ethyl-2-methyl-1,3-hexadiene	1421	MS/KI	0.11	0.00 ^d^	0.01 ^d^	0.04 ^d^	0.22 ^bc^	0.16 ^bcd^	0.25 ^ab^	0.39 ^a^	0.06 ^cd^	<0.0001
Ketones												
4-methyl-2-hexanone			0.10	0.01 ^c^	0.05 ^c^	0.07 ^c^	0.31 ^b^	0.41 ^ab^	0.35 ^ab^	0.49 ^a^	0.06 ^c^	<0.0001
1-octen-3-one	1290	MS/KI	0.28	0.00 ^d^	0.06 ^d^	0.12 ^cd^	0.18 ^cd^	0.51 ^bc^	0.73 ^ab^	1.00 ^a^	0.30 ^cd^	<0.0001
2,3-Octanedione	1323	MS/KI	0.04	0.06 ^ab^	0.10 ^a^	0.07 ^ab^	0.07 ^ab^	0.07 ^ab^	0.10 ^a^	0.02 ^b^	0.01 ^b^	<0.0001
6-methyl-5-hepten-2-one	1342	MS/KI	0.02	0.00 ^c^	0.00 ^bc^	0.00 ^c^	0.02 ^bc^	0.03 ^b^	0.08 ^a^	0.09 ^a^	0.07 ^a^	<0.0001
3-octen-2-one	1388	MS/KI	0.71	0.05 ^d^	0.06 ^d^	0.09 ^d^	0.67 ^cd^	1.48 ^abc^	1.75 ^ab^	2.14 ^a^	0.67 ^bcd^	<0.0001
6-methoxy-2-hexanone,		MS	0.12	0.14 ^bc^	0.26 ^abc^	0.21 ^bc^	0.41 ^a^	0.29 ^ab^	0.24 ^bc^	0.29 ^ab^	0.09 ^c^	0.0007
2-decanone	1493	MS/KI	0.04	0.02 ^c^	0.04 ^ab^	0.03 ^ab^	0.08 ^a^	0.07 ^ab^	0.05 ^ab^	0.07 ^ab^	0.02 ^c^	0.0008
3,5-octadien-2-one	1516	MS/KI	1.49	0.08 ^c^	0.15 ^c^	0.15 ^c^	1.20 ^bc^	3.84 ^a^	2.99 ^ab^	4.42 ^a^	1.50 ^bc^	<0.0001
4-hexen-2-one		MS	17.91	20.63 ^c^	38.08 ^abc^	31.11 ^bc^	59.45 ^a^	48.16 ^ab^	37.01 ^abc^	47.65 ^ab^	14.26 ^c^	<0.0001
2-Undecanone	1592	MS/KI	3.37	3.29 ^bc^	6.15 ^abc^	5.30 ^abc^	8.76 ^a^	6.40 ^ab^	6.42 ^ab^	2.35 ^bc^	1.07 ^c^	<0.0001
Acetophenone	1600	MS/KI	0.09	0.01 ^c^	0.03 ^c^	0.05 ^c^	0.40 ^a^	0.26 ^b^	0.30 ^ab^	0.32 ^ab^	0.07 ^c^	<0.0001
2-pentadecanone	2011	MS/KI	0.41	0.70 ^a^	0.74 ^a^	0.52 ^ab^	0.55 ^ab^	0.40 ^b^	0.12 ^d^	0.21 ^c^	0.24 ^c^	0.019
Alcohols												
1-hexanol	1362	MS/KI	0.48	0.01 ^b^	0.02 ^b^	0.09 ^b^	0.88 ^a^	0.57 ^ab^	0.56 ^ab^	1.17 ^a^	0.16 ^b^	<0.0001
2-butoxyethanol	1403	MS/KI	1.08	0.03 ^c^	0.28 ^bc^	0.39 ^bc^	0.50 ^bc^	1.73 ^ab^	2.57 ^a^	2.96 ^a^	0.25 ^bc^	<0.0001
1-octen-3-ol	1459	MS/KI	2.29	0.02 ^d^	0.39 ^d^	1.31 ^d^	5.84 ^ab^	4.64 ^bc^	6.79 ^ab^	8.12 ^a^	2.28 ^cd^	<0.0001
1-heptanol	1467	MS/KI	0.34	0.01 ^e^	0.03 ^de^	0.14 ^de^	0.51 ^bcd^	0.82 ^ab^	0.73 ^bc^	1.23 ^a^	0.24 ^cde^	<0.0001
1-octanol	1565	MS/KI	0.36	0.00 ^d^	0.04 ^d^	0.13 ^cd^	0.59 ^bc^	0.77 ^ab^	0.74 ^ab^	1.26 ^a^	0.24 ^bcd^	<0.0001
2-undecanol	1706	MS/KI	0.28	0.01 ^d^	0.05 ^cd^	0.08 ^cd^	0.15 ^cd^	0.45 ^bc^	0.62 ^ab^	0.92 ^a^	0.18 ^cd^	<0.0001
2-methyl-1-decanol	1803	MS/KI	0.27	0.01 ^b^	0.02 ^b^	0.03 ^b^	0.12 ^b^	0.21 ^b^	0.29 ^ab^	0.62 ^a^	0.06 ^b^	<0.0001
1-(2-butoxyethoxy)ethanol	1800	MS/KI	0.22	0.04 ^d^	0.14 ^cd^	0.14 ^cd^	0.40 ^bc^	0.26 ^bcd^	0.55 ^ab^	0.83 ^a^	0.09 ^cd^	<0.0001
Benzenemethanol	1865	MS/KI	0.08	0.26 ^a^	0.27 ^a^	0.19 ^ab^	0.12 ^bc^	0.12 ^bc^	0.04 ^c^	0.07 ^c^	0.07 ^bc^	<0.0001
Phenyl ethyl alcohol	1925	MS/KI	0.71	0.06	0.21	0.72	0.17	0.22	0.03	0.08	0.03	0.436
Phenol	2000	MS/KI	0.37	1.60 ^a^	1.05 ^ab^	0.76 ^bc^	0.43 ^cd^	0.27 ^cd^	0.07 ^d^	0.11 ^d^	0.09 ^d^	<0.0001
2-octen-1-ol		MS	5.12	3.08 ^b^	4.14 ^b^	3.70 ^b^	6.21 ^b^	7.94 ^ab^	8.70 ^ab^	14.11 ^a^	2.51 ^b^	<0.0001
Acids												
2-methyl-anhydride pentanoic acid		MS	1.81	0.17 ^c^	0.35 ^bc^	0.72 ^bc^	0.94 ^bc^	1.61 ^bc^	3.11 ^bc^	4.50 ^a^	0.69 ^bc^	<0.0001
Pentadecanoic acid	2822	MS/KI	2.63	0.44 ^b^	0.87 ^b^	0.61 ^b^	1.76 ^b^	6.71 ^a^	1.94 ^b^	3.05 ^ab^	3.30 ^ab^	<0.0001
Heptanoic acid	1971	MS/KI	1.36	0.70	1.35	1.22	1.30	2.26	0.30	0.50	0.40	0.046
Octanoic Acid	2048	MS/KI	6.77	3.65 ^b^	9.48 ^ab^	10.36 ^ab^	9.37 ^ab^	13.78 ^a^	4.85 ^ab^	7.35 ^ab^	8.98 ^ab^	0.06
Nonanoic acid	2128	MS/KI	5.24	5.00	8.44	5.14	4.92	2.95	0.61	1.24	0.90	0.044
decanoic acid	2296	MS/KI	6.63	0.96 ^c^	1.90 ^c^	5.35 ^c^	17.56 ^b^	45.63 ^a^	36.17 ^a^	45.62 ^a^	37.22 ^a^	<0.0001
9-Decenoic acid	1348	MS/KI	0.95	0.08 ^d^	0.18 ^d^	0.67 ^d^	2.57 ^c^	5.37 ^a^	3.95 ^bc^	5.54 ^a^	4.41 ^ab^	<0.0001
n-Hexadecanoic acid	2910	MS/KI	8.30	0.80 ^c^	0.92 ^c^	0.75 ^c^	10.69 ^bc^	28.76 ^a^	24.46 ^a^	32.55 ^a^	21.08 ^ab^	<0.0001
Dodecanoic acid	2517	MS/KI	1.59	0.42 ^d^	0.33 ^d^	0.83 ^d^	3.73 ^c^	10.60 ^ab^	8.87 ^b^	11.32 ^a^	8.27 ^b^	<0.0001
Ohers												
1,5-Diphenyl-3-methylthio-1,2,4-triazole		MS	0.03	0.02 ^c^	0.09 ^a^	0.04 ^bc^	0.05 ^bc^	0.05 ^bc^	0.08 ^ab^	0.08 ^ab^	0.02 ^c^	<0.0001
2-pentylfuran	1244	MS/KI	1.10	1.26 ^bc^	3.16 ^a^	2.55 ^ab^	2.09 ^abc^	2.94 ^a^	0.90 ^c^	1.26 ^bc^	0.72 ^c^	<0.0001
2(3H)dihydro-5-penthylfuranone		MS	2.13	0.73 ^bc^	1.35 ^abc^	0.86 ^bc^	3.78 ^ab^	4.49 ^a^	0.57 ^c^	1.71 ^abc^	0.42 ^c^	0.0002

^a^ Kovat’s index (KI); ^b^ Identification (ID) was carried out by comparing each mass spectrum in NIST 05 or Wiley 7 databases (MS); matching with reported Kovat’s indices (KI); ^c^ Root mean square error; Different letters (a, b, c, d, e) within the same chemical family indicate significant differences (*p* < 0.05) among maturing times.

**Table 4:** Volatile compounds of subcutaneous fat of Toscano dry-cured ham selected by stepwise discriminant analysis. Canonical discriminant analysis scores (Can1) were used to separate low maturing classes (LMC) and high maturing classes (HMC).

Subcutaneous Fat Samples			
	Chemical Family	Can1	Sensory Descriptors ^a^
1,1-diethoxyhexane	Hydrocarbon	−0.82	Cognac, pear, floral, hyacinth, apple, fruity ^1^
3-methyl-ethyl ester butanoic acid	Ester	0.03	Strong, fruity, vinous, apple-like
2,4-dimethylbenzaldehyde	Aldehyde	0.13	Mild, sweet, bitter-almond
Butanoic acid, ethyl ester	Ester	0.45	Fruity odor with pineapple undertone and sweet
Dodecanoic acid	Acid	2.42	Fatty, creamy, cheese-like, waxy

**^a^** As reported in Burdock, G.A, 2010 [31], except for: ^1^ Based on online databases www.thegoodscentscompany.com.

**Table 5:** Volatile compounds of subcutaneous fat of Toscano dry-cured ham selected by stepwise discriminant analysis. Canonical discriminant analysis scores (Can1, Can2, and Can3) were used to separate hams belonging to different seasoning lengths (S12, S14, S16, S18).

Subcutaneous Fat Samples					
	Chemical Family	Can1	Can2	Can3	Sensory Descriptors ^a^
1,1-diethoxyhexane	Hydrocarbon	−0.75	−0.44	−0.03	Cognac, pear, floral, hyacinth, apple, fruity ^1^
Pentanoic acid, ethyl ester	Ester	0.48	0.12	0.74	Fruity, apple-like
4-methyl-2-hexanone	Ketone	2.17	0.93	0.64	Fruity ^2^
2,4,4-trimethylhexane	Hydrocarbon	0.37	−1.13	0.69	-
2,3-octanedione	Ketone	−3.26	0.20	0.55	Green, spicy, cilantro, fatty, leafy, cortex, herbal, warmerd-over
Formic Acid, ethyl ester	Ester	−0.10	1.18	−0.88	Pungent, rum-like, pineapple
6-methoxy-2-hexanone	Ketone	−0.76	1.03	0.63	Fruity e spicy ^3^
Decanal	Aldehyde	1.07	−0.06	−0.30	Sweet, waxy, floral, citrus, fatty
Acetic acid, ethenyl ester	Ester	0.88	0.04	−0.50	Wine, fruity ^4^
dihydro-5-penthyl-2(3H) furanone	Furanone	0.00	0.85	0.57	Coconut and fatty
Hexadecanoic acid, ethyl ester	Ester	0.81	0.45	0.02	Mild, waxy sweet
Undecanoic acid, methyl ester	Ester	−0.07	−1.22	0.02	Fatty, waxy fruity ^1^
					
Proportion of explained variation		0.56	0.31	0.13	

**^a^** As reported in Burdock, G.A, 2010 [31], except for: ^1^ Based on online databases www.thegoodscentscompany.com; ^2^ Reale et el., 2019 [68]; ^3^ Luna et al., 2006 [19]; ^4^ Lin et al., 2014 [69].

## Data Availability

The data presented in this study are available on request from the corresponding author.
